# Translocation through the Conjugative Type IV Secretion System Requires Unfolding of Its Protein Substrate

**DOI:** 10.1128/JB.00615-17

**Published:** 2018-02-23

**Authors:** Martina Trokter, Gabriel Waksman

**Affiliations:** a Institute of Structural and Molecular Biology, Department of Biological Sciences, Birkbeck, London, United Kingdom; Michigan State University

**Keywords:** T4SS, transport, conjugation, secretion systems, type 4 secretion systems, unfolding

## Abstract

Bacterial conjugation, a mechanism of horizontal gene transfer, is the major means by which antibiotic resistance spreads among bacteria (1, 2). Conjugative plasmids are transferred from one bacterium to another through a type IV secretion system (T4SS) in the form of single-stranded DNA covalently attached to a protein called relaxase. The relaxase is fully functional both in a donor cell (prior to conjugation) and recipient cell (after conjugation). Here, we demonstrate that the protein substrate has to unfold for efficient translocation through the conjugative T4SS. Furthermore, we present various relaxase modifications that preserve the function of the relaxase but block substrate translocation. This study brings us a step closer to deciphering the complete mechanism of T4SS substrate translocation, which is vital for the development of new therapies against multidrug-resistant pathogenic bacteria.

**IMPORTANCE** Conjugation is the principal means by which antibiotic resistance genes spread from one bacterium to another (1, 2). During conjugation, a covalent complex of single-stranded DNA and a protein termed relaxase is transported by a type IV secretion system. To date, it is not known whether the relaxase requires unfolding prior to transport. In this report, we use functional assays to monitor the transport of wild-type relaxase and variants containing unfolding-resistant domains and show that these domains reduce conjugation and protein transport dramatically. Mutations that lower the free energy of unfolding in these domains do not block translocation and can even promote it. We thus conclude that the unfolding of the protein substrate is required during transport.

## INTRODUCTION

Bacteria have evolved a diversity of specialized secretion systems that allow them to translocate macromolecules across the cell envelope (3). Among them, the type IV secretion system (T4SS) is the most versatile (4). T4SSs mediate the transfer of DNA and protein substrates across the cell envelope. The largest and most widely distributed of the T4SS subfamilies are conjugation systems (5).

Conjugation is a major mechanism of horizontal gene transfer (6, 7). It is a process by which one bacterium, the donor, transfers genetic material to another bacterium, the recipient, in a contact-dependent manner (8). Thus, conjugation is the major means by which antibiotic resistance genes spread among bacterial populations (1, 2). Conjugation occurs widely among Gram-negative bacteria, Gram-positive bacteria, and even some archaea (9).

Many plasmids and integrative and conjugative elements (ICEs), so-called “mobile elements,” undergo conjugation (10). Many of these mobile genetic elements are self-transmissible: they encode the entire machinery necessary for their transfer into recipient cells. Proteins necessary for conjugation assemble into two complexes: a DNA-processing complex called the relaxosome and a complex responsible for transfer, the T4SS. The relaxosome is an assembly of a protein called relaxase and a few accessory proteins that bind a specific DNA sequence called *oriT* (origin of transfer) to form a nucleoprotein complex (11). The T4SS is a large (3 to 4 MDa) protein complex consisting of a transport apparatus that spans the bacterial cell envelope (12), a pilus that extends from the cell surface (13) and mediates contact between cells (14), and a type IV coupling protein (T4CP) (15, 16) that recruits the relaxosome to the secretion channel.

The general mechanism of conjugation is still poorly understood, but some steps are known (11, 17). The relaxase encoded by a number (but not all) of plasmids is an enzyme that often has two activities, transesterase/nicking activity and helicase activity. The transesterase nicks the plasmid DNA strand destined for transfer (T-strand) at a specific position within *oriT*, called *nic*, and remains covalently attached through a catalytic tyrosine to the 5′-phosphate end of the cleaved strand. The relaxase and accessory proteins carry translocation signals for recruitment of the transfer intermediate to the T4SS via T4CP. Upon contact with a recipient cell, the substrate (the relaxase covalently attached to the T-strand) is transported into the recipient cell in an ATPase-dependent manner. During the translocation process, the T-strand is unwound from its complementary strand by a second copy of the relaxase, the helicase activity of which motors the T-strand through the T4SS, presumably assisted by some of the T4SS ATPases (18). In the recipient cell, the relaxase molecule that has passed through the system may recircularize the T-strand, and the complementary strand is synthesized (17).

R388 is one of the best-studied conjugative plasmids that belong to a broad-host-range group of plasmids (19, 20). Proteins essential for conjugation are encoded within two separate gene clusters. One cluster contains *oriT* and genes encoding the accessory protein TrwA, the T4CP protein TrwB, and the relaxase TrwC, whereas the other cluster encodes 11 T4SS proteins, TrwN to TrwD (TrwN-TrwD), homologs of the VirB1 to VirB11 (VirB1-VirB11) proteins of the prototypical T4SS from Agrobacterium tumefaciens, the VirB/D4 system (21, 22).

The TrwC relaxase is a 107-kDa protein composed of two domains: an N-terminal transesterase (also termed “relaxase”) domain (approximately 1 to 300 residues) and a C-terminal helicase domain (approximately 300 to 966 residues). High-resolution crystal structures of the TrwC relaxase domain in complex with oligonucleotides containing TrwC binding and/or nicking sites revealed details of DNA binding site recognition and a nicking mechanism by TrwC (23, 24). Nucleophilic attack of the *nic* site by the catalytic tyrosine, Tyr18 (25), generates a phosphotyrosine bond between the cleaved T-strand 5′-phosphate and the Tyr residue in the relaxase. The C terminus of TrwC (residues 796 to 802) contains a translocation signal for recruitment by the T4SS machinery (26). The helicase domain contains a 5′–3′ helicase activity (27). Once in the recipient cell, the helicase domain is thought to track in the 5′ to 3′ direction along the T-strand in order to position it correctly for the termination rejoining step (28). It is also thought to be responsible for the unwinding of the T-strand during conjugation. In this case, the T-strand-unwinding TrwC molecule would have to be distinct from the translocated one (18).

The fact that the relaxase has to pass through the T4SS raises the question of whether the relaxase is transported in a folded or unfolded state through the T4SS channel. Among other types of bacterial secretion systems, some are known to transport folded substrates (such as type 2 secretion systems [29] and the chaperone-usher pathway [30, 31]), whereas some can translocate only unfolded substrates (e.g., type 1 secretion systems [32] and type 3 secretion systems [33, 34]). The negative-stain electron microscopy structure of the TrwM/VirB3-TrwE/VirB10 complex from R388 T4SS has recently revealed the T4SS architecture (12). However, the internal channel, the dimensions of which might give a clue on the folding state of the substrate during transport, has not yet been identified. In this report, we have studied the requirements for the substrate translocation by the R388 conjugative T4SS. We show that the T4SS substrates have to be unfolded in order to be translocated into recipient cells.

## RESULTS

The overall aim of this study is to investigate whether transport of the relaxase requires unfolding of the protein. In order to answer this question, our strategy was to fuse unfolding-resistant proteins or protein modules of various sizes to TrwC and test if they can be transported into recipient cells. We first sought to test this by directly monitoring the transport of the protein itself during conjugation using the previously described Cre recombinase reporter assay for translocation (CRAfT [35, 36]).

### Establishing a translocation assay of the TrwC relaxase based on the CRAfT assay.

Briefly, the recipient strain contains a chloramphenicol resistance gene interrupted by a tetracycline resistance cassette flanked by *loxP* sites. Therefore, the strain is tetracycline resistant, but upon Cre recombination, it becomes tetracycline sensitive and chloramphenicol resistant. The transfer of Cre recombinase-substrate fusion can be measured by measuring the change in antibiotic resistance of recipient cells upon conjugation. We used a two-plasmid system composed of a plasmid containing R388 *oriT*, termed pRSF-oriT, and a plasmid encoding relaxosome components (TrwA, TrwB, and TrwC) and T4SS, termed pBAD-ABC-T4SS. This two-plasmid system typically resulted in 60 to 80% of the recipients acquiring the *oriT* plasmid (transconjugants) in our conjugation assay.

**FIG 1:**
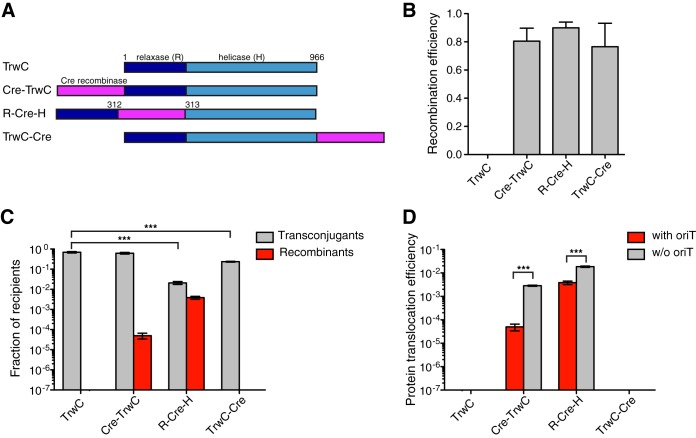
Establishing a Cre recombinase reporter assay for translocation of R388 T4SS substrates. (A) Scheme of TrwC constructs with Cre recombinase fusion at different positions. (B) Recombination efficiency of Cre recombinase fused to the TrwC N terminus (Cre-TrwC), TrwC C terminus (TrwC-Cre), and between the relaxase and helicase domains (R-Cre-H) when expressed in recipient cells. Values represent the mean ± standard error of the mean (SEM) of the results from four experiments. Unpaired *t* test showed no significant difference between recombination efficiencies of Cre-TrwC, R-Cre-H, and TrwC-Cre (*P* > 0.05). (C) Conjugation and protein translocation efficiency of cells carrying pRSF-oriT and pBAD-ABC-T4SS plasmid encoding modified TrwC, as indicated. The efficiencies are expressed as a fraction of recipient cells that acquired pRSF-oriT plasmid (transconjugants) and TrwC fusion protein (recombinants), respectively. Values represent the mean ± SEM of the results from three experiments. Statistically significant differences (unpaired *t* test) between wild-type and Cre fusion construct conjugation frequencies are indicated. ***, *P* ≤ 0.001. (D) The comparison of TrwC translocation in the presence (red bars) or absence (gray bars) of pRSF-oriT plasmid. The protein translocation efficiencies are expressed as a fraction of recipient cells that acquired the indicated TrwC fusion protein. Values represent the mean ± SEM of the results from four experiments. Statistically significant differences (unpaired *t* test) between translocation frequencies in the presence or absence of *oriT* are indicated. ***, *P* ≤ 0.001.

We first fused the Cre recombinase at different locations within the TrwC substrate and tested which construct retains functionality (both in plasmid conjugation and Cre recombination). Cre recombinase was fused to the TrwC N terminus (termed Cre-TrwC), TrwC C terminus (termed TrwC-Cre), and between the relaxase and helicase domains (termed R-Cre-H) (Fig. 1A). Given the fact that the first methionine (Met1) of the N-terminal relaxase domain is located at the center of the relaxase structure and participates in DNA binding, together with Leu2, His4, Met5, and Val6 (24), there was a possibility that the N-terminal fusion would abolish *oriT* binding and/or nicking and, therefore, conjugation. Nevertheless, TrwC transfer without DNA transfer has previously been observed (37); thus, we proceeded with making and testing all three Cre fusions of TrwC, even the N-terminal fusion.

Cre recombinase retained functionality in all three fusions (Fig. 1B). Surprisingly, the conjugation level of all three fusions was high, even for the N-terminal fusion; however, protein transfer was detected only in the case of Cre-TrwC and R-Cre-H (Fig. 1C). Since TrwC-Cre is not transported into the recipient cells at detectable levels, the high conjugation efficiency observed with this construct is most likely due to a fraction of TrwC lacking the C-terminal Cre fusion, a result of proteolytic degradation within the cell.

**FIG 2:**
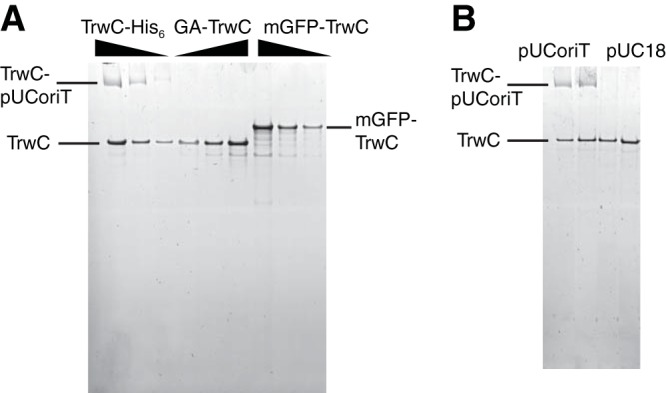
N-terminal fusions abolish TrwC nicking of *oriT*. (A) Sypro Ruby-stained SDS gel showing purified TrwC-His_6_, GA-TrwC (glycine-alanine-TrwC), and mGFP-TrwC free or covalently bound to pUC-oriT. Constructs with 40, 80, or 160 nM TrwC were incubated at 37°C for 30 min with 40 nM pUC-oriT before the nicking reaction was stopped by adding SDS sample buffer and EDTA and heating at 95°C. (B) TrwC-His_6_ nicks *oriT* specifically. TrwC-His_6_ at 50 nM (lanes 1 and 3) or 100 nM (lanes 2 and 4) was incubated with either pUC-oriT or pUC18 plasmid (20 nM), as indicated, at 37°C for 30 min before the reaction was stopped by adding SDS sample buffer and EDTA and heating at 95°C.

Cre-TrwC displays a high level of conjugation, indicating that N-terminal fusions of TrwC might be active in DNA transport after all. We therefore tested purified TrwC with and without N-terminal fusions in an *oriT* nicking assay. As shown in Fig. 2, whereas TrwC-His_6_ formed a covalent linkage with the pUC-oriT plasmid, we did not detect any monomeric green fluorescent protein-TrwC (mGFP-TrwC), or even GA-TrwC (TrwC with an additional N-terminal glycine and alanine residue), covalently bound to *oriT*. Since even only two additional N-terminal residues can abolish TrwC activity, the source of TrwC protein that is functional in conjugation is most likely wild-type TrwC coexpressed starting from the original start codon. The linker sequence between Cre and TrwC that lies just upstream of the TrwC start codon is rich in GG nucleotides, possibly acting as a ribosome-binding site. In conclusion, both N-terminal and C-terminal fusions abolish TrwC transport and/or activity.

Unlike the N-terminal and C-terminal Cre fusions, which can both apparently yield a Cre-less wild-type version of the protein (see above), the internal Cre fusion construct cannot undergo modifications yielding wild-type TrwC. Indeed, separated relaxase and helicase domains that could arise by proteolytic degradation are not functional (27, 38). Therefore, the measured conjugation efficiency of R-Cre-H reflects the activity of the full-length protein. R-Cre-H also showed almost two orders of magnitude higher level of protein translocation in the CRAfT experiment than Cre-TrwC (Fig. 1C). Since Cre-TrwC is not functional in *oriT* nicking, the protein is in this case transported without being attached to the T-strand, as described by Draper et al. (37). In order to test if *oriT* binding is the reason for higher protein translocation of R-Cre-H, we repeated the CRAfT assay but this time excluding the pRSF-oriT plasmid. Surprisingly, we detected higher protein transport in the absence of the *oriT* plasmid than in its presence (Fig. 1D). This indicates that the Cre recombination in recipient cells might be affected by the presence of the T-strand, possibly due to competition with the TrwC helicase activity along the T-strand.

### Substrate unfolding is a prerequirement for translocation through T4SS.

**FIG 3:**
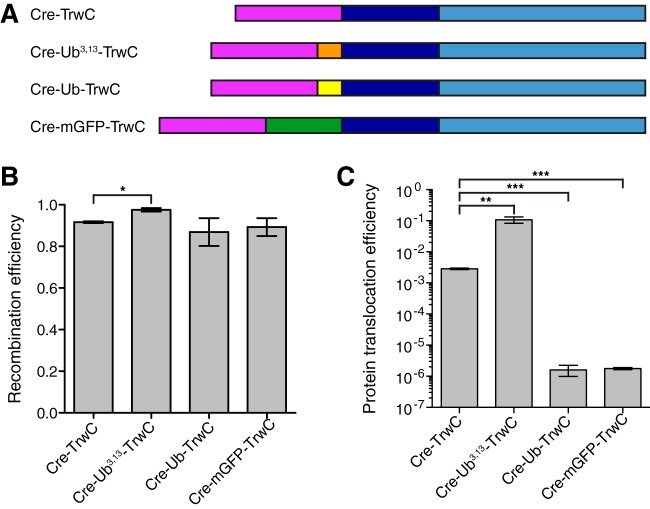
TrwC unfolding is necessary for its translocation through the T4SS. (A) Scheme of TrwC constructs with N-terminal Cre recombinase followed by an unfoldable (Ub^3,13^) or unfolding-resistant (Ub and mGFP) fusion. (B) Recombination efficiency of Cre recombinase fused to the N terminus of the indicated protein followed by TrwC when expressed in recipient cells. Values represent the mean ± SEM of the results from three experiments. Statistically significant differences (unpaired *t* test) between recombination efficiencies of Cre-TrwC and constructs with an additional fusion are indicated. *, *P* ≤ 0.05; **, *P* ≤ 0.01; ***, *P* ≤ 0.001. (C) Protein translocation efficiency of cells carrying pBAD-ABC-T4SS plasmid encoding modified TrwC, as indicated. Values represent the mean ± SEM of the results from four experiments. Statistically significant differences (unpaired *t* test) between protein translocation efficiencies of Cre-TrwC and constructs with an additional fusion are indicated. *, *P* ≤ 0.05; **, *P* ≤ 0.01; ***, *P* ≤ 0.001.

Next, we chose the N-terminal fusions to test the extent of substrate unfolding required for its transport through the T4SS. We fused a set of “unfoldable” (here meaning “that can be unfolded”) and unfolding-resistant (that cannot be unfolded) proteins between Cre and TrwC (Fig. 3A) and tested if they can be transported into recipient cells. With Cre recombinase positioned at the very N terminus and TrwC translocation signal at the very C terminus, these constructs allow us to detect the transport of only full-length proteins. Ubiquitin (Ub; 8 kDa) and GFP (27 kDa) have been reported to be resistant to unfolding, whereas the ubiquitin mutant Ub^I3G,I13G^ (Ub^3,13^) served as the unfoldable protein control (34, 39). We first tested the Cre recombinase activity of these constructs. Cre recombinase was functional in all fusions tested (Fig. 3B).

When tested in the CRAfT assay, the protein construct containing the unfoldable fusion, the ubiquitin mutant (Ub^3,13^), was transported into recipient cells at very high levels (∼10% of the recipient cells underwent recombination) (Fig. 3C). In comparison, its wild-type ubiquitin counterpart was transported at a level of about five orders of magnitude lower, similar to the level of the construct containing mGFP. These data clearly demonstrate that the conjugative T4SS substrate, the relaxase protein, has to be unfolded in order to pass through the T4SS channel.

### Relaxase constructs that block substrate translocation in the native plasmid.

**FIG 4:**
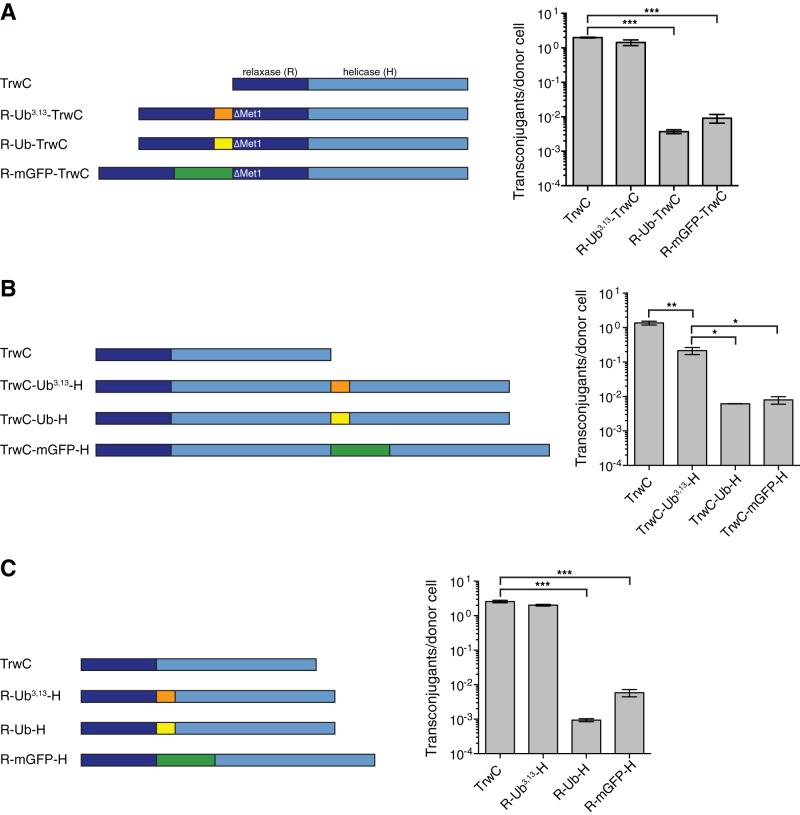
Conjugation of TrwC fusions in native R388 plasmid. (A) Duplicated relaxase domain recovers *oriT* nicking activity of TrwC with N-terminal fusion. Left, scheme of TrwC constructs with duplicated relaxase domain and indicated fusion protein (termed R-X-TrwC, where X represents Ub^3,13^, Ub, or mGFP). Right, conjugation efficiency of cells carrying R388 plasmid encoding modified TrwC, as indicated. Values represent the mean ± SEM of the results from three experiments. Statistically significant differences (unpaired *t* test) between wild-type and fusion construct conjugation frequencies are indicated. ***, *P* ≤ 0.001. (B) Conjugation efficiency of TrwC-fusion-helicase constructs. Left, scheme of TrwC constructs with indicated fusion protein and duplicated helicase domain (termed TrwC-X-H, where X represents Ub^3,13^, Ub, or mGFP). Right, conjugation efficiency of cells carrying R388 plasmid encoding modified TrwC, as indicated. Values represent the mean ± SEM of the results from three experiments. Statistically significant differences (unpaired *t* test) between conjugation frequencies of indicated pairs of constructs are shown. *, *P* ≤ 0.05; **, *P* ≤ 0.01. (C) TrwC relaxase and helicase domains retain their functionality after being separated by an internal fusion. Left, scheme of TrwC constructs with indicated internal fusion (termed R-X-H, where X represents Ub^3,13^, Ub, or mGFP). Right, conjugation efficiency of cells carrying R388 plasmid encoding modified TrwC, as indicated. Values represent the mean ± SEM of the results from three experiments. Statistically significant differences (unpaired *t* test) between wild-type and fusion construct conjugation frequencies are indicated. ***, *P* ≤ 0.001.

The experiments described above were conducted using an artificial two-plasmid system reporting on protein transport to the recipient cell. We sought next to monitor the nucleoprotein substrate transport through conjugative T4SS using the native R388 plasmid. We also sought to expand the range of fusion proteins probed. For this purpose, it was necessary to generate a modified protein substrate that is fully functional. We therefore generated three different sets of relaxase constructs and tested their functionality. For these experiments, we directly modified TrwC in the wild-type R388 plasmid. Since N-terminal fusions abolish *oriT* nicking activity, we tested if duplicating the relaxase domain in front of the N-terminal fusion can recover relaxase activity (Fig. 4A, left). In order to avoid expression of the wild-type TrwC from its original start codon, the first methionine in the second relaxase domain was deleted. Indeed, the addition of the relaxase domain in front of the unfoldable ubiquitin fusion resulted in wild-type levels of relaxase activity (Fig. 4A, right). The high conjugation efficiency of this construct is not the result of proteolysis of mutant ubiquitin (see Fig. S1 in the supplemental material). Replacing an unfoldable fusion with an unfolding-resistant one efficiently blocks the substrate transport, resulting in more than two orders of magnitude lower conjugation efficiency (Fig. 4A, right).

Equivalently, an attempt was made to recover the activity of C-terminal fusions by adding another helicase domain (H) to the C terminus of a TrwC-fusion (Fig. 4B, left). In this case, the duplication of the helicase domain did not completely restore the wild-type level of relaxase activity (Fig. 4B, right), potentially due to the suboptimal position of the translocation signal (e.g., too large a distance between the relaxase and the functional translocation signal). The difference in the conjugation efficiencies between the unfolding-resistant and unfoldable ubiquitin construct is lower than for the constructs with the duplicated relaxase. The observed conjugation efficiency of TrwC-Ub/mGFP-H constructs might be a result of either coexpression of the wild-type TrwC (as a result of either degradation and/or premature translation termination) or suboptimal folding of ubiquitin and mGFP at the TrwC C terminus.

Finally, we tested the activity of TrwC containing internal fusions between its relaxase and helicase domains (Fig. 4C, left). Internal fusion with Ub^3,13^ retains a wild-type level of activity (Fig. 4C, right). Unfolding-resistant (Ub or mGFP) fusions efficiently blocked substrate transport, with conjugation efficiencies of more than three orders of magnitude lower than that of its unfoldable counterpart.

We note that in all unfolding-resistant variants tested, a very low residual level of translocation/transfer is observed, likely due to a small fraction of these proteins being less resistant to unfolding due to defects in their folded state.

### Conclusion.

Secretion in bacteria is a critical process in pathogenesis and interbacterial competition in many bacterial pathogens. Bacteria have evolved a diversity of specialized secretion systems to export a wide range of substrates, including small molecules, proteins, and DNA, across the cell envelope (3). Some secretion systems, such as the T2SS (29) and the chaperone-usher pathway (30, 31), transport fully folded protein substrates, whereas some can transport only unfolded substrates (e.g., T1SS [32] and T3SS [33, 34]). In this report, we demonstrate that conjugative T4SS substrates, relaxases, have to undergo unfolding in order to be transported through the T4SS channel. A comparison of translocation frequencies of unfolding-resistant TrwC fusions and their unfoldable counterparts clearly demonstrated that both TrwC alone and TrwC covalently attached to the T-strand are transported into recipient cells in an unfolded state. In that respect, conjugative T4SSs work in a manner similar to that of effector only-transporting T4SSs, such as that of the bacterial pathogen Legionella pneumophila (40).

T1SS substrates fold upon binding of calcium, which is low in the bacterial cytosol but high in the extracellular space; therefore, it is generally assumed that T1SS substrates adopt their folded conformation only after secretion into the extracellular space. Conjugative relaxases are clearly folded prior to their translocation, as they are fully functional when expressed in a bacterial cell. Which enzyme/unfoldase is responsible for T4SS substrate unfolding remains unclear. In case of T3SS, a dedicated hexameric ATPase has a critical function in substrate recognition and unfolding in an ATP-dependent manner (33). T4SS, on the other hand, is energized by three distinct hexameric ATPases: VirB4, VirB11, and T4CP. T4CP is an integral membrane protein that interacts with the relaxase and accessory proteins and recruits the transfer intermediate to the T4SS translocation machinery. T4CP ATPase activity is not required for the recruitment (41). However, the activities of all three ATPases are essential for nucleoprotein transport (41). VirB4 is an integral part of the T4SS inner membrane complex (IMC) and is located mainly in the cytoplasm. VirB11 is a cytoplasmic ATPase that interacts with T4CP and VirB4. Apart from being essential for substrate transport, VirB4 and VirB11 are required for T4SS pilus assembly. Due to their several roles that are essential for T4SS function, it is difficult to predict which ATPase is responsible for substrate unfolding.

The CRAfT experiments performed here also revealed that an N-terminal fusion might abolish a relaxase function (Fig. 1). TrwC *oriT* nicking activity was sensitive to the addition of only two amino acids at its N terminus (Fig. 2). The crystal structure of the TrwC relaxase domain in complex with its cognate DNA at *oriT* showed that first five TrwC residues participate in binding DNA (24). The N-terminal methionine alone forms multiple interactions with DNA. Its side chain is trapped in a hydrophobic cage formed by a sharp U-turn of the T-strand DNA, whereas its amino group forms a hydrogen bond with the DNA. Therefore, the addition of any amino acids to the relaxase N terminus might perturb *oriT* binding and result in an inactive protein.

Finally, we showed here that although the terminal fusions abolish the relaxase activity, it is possible to modify the relaxase in different ways in order to preserve the functionality of its domains. Placing a fusion internally or duplicating a domain can recover a relaxase activity, and unfolding-resistant fusions can be used to efficiently block the substrate transport. This will be particularly important for deciphering the complete mechanism of T4SS substrate translocation. T4SS substrates modified with unfolding-resistant fusions at appropriate locations could be used as a tool to efficiently trap the substrate during translocation. Structural studies of the T4SS in complex with the substrate trapped within will allow the substrate translocation path and T4SS conformational changes necessary for translocation to be defined. Deciphering the details of the T4SS translocation mechanism will be vital to facilitate the development of new therapies against multidrug-resistant pathogenic bacteria.

## MATERIALS AND METHODS

### Molecular biology.

The *oriT* and expression plasmids used in this study are described in Table S1 in the supplemental material. Primer sequences are shown in Table S2. DNA fragments used for cloning were amplified using Phusion high-fidelity DNA polymerase (NEB), according to the manufacturer's instructions. Restriction enzymes were obtained from NEB. Unless otherwise stated, the antibiotic concentrations used were as follows: kanamycin (Km), 30 μg/ml; ampicillin (Amp), 100 μg/ml; tetracycline (Tc), 10 μg/ml; chloramphenicol (Cm), 10 μg/ml; streptomycin (Sm), 25 μg/ml; and trimethoprim (Tmp), 10 μg/ml.

pRSF-oriT was generated by ligation (rapid DNA ligation kit; Roche) of amplified *oriT* and pRSFDuet-1 vector digested with the BssHII restriction enzyme. pUC-oriT was generated by ligation of amplified *oriT* and pUC18 vector digested with HindIII-HF and SacI-HF restriction enzymes.

Unless otherwise stated, all plasmids used in this study were generated by seamless cloning using the In-Fusion HD cloning kit (Clontech). In most cases, the constructs were generated by fusing two PCR fragments. In some cases (described below), the constructs were generated by fusing a PCR fragment and a linearized vector. pBAD-trwN/virB1-trwE/virB10_Strep_-trwD/virB11 was cloned by amplifying *trwD* or *virB11* and inserting it into the pBAD-trwN/virB1-trwE/virB10_Strep_ plasmid (12), which was linearized using the BstBI restriction enzyme. pBAD-ABC-T4SS was cloned by amplifying *trwABC*_His_ and inserting it into the pBAD-trwN/virB1-trwE/virB10_Strep_-trwD/virB11 plasmid, which was linearized using the NcoI restriction enzyme. All pBAD-ABC-T4SS constructs encoding modified TrwC were cloned in the same way (by amplifying ABC and inserting into the linearized pBAD-trwN/virB1-trwE/virB10_Strep_-trwD/virB11 plasmid).

TrwC internal fusions were inserted into an unstructured region between the relaxase and helicase domains (between residues 312 and 313). The TrwC secondary structure was predicted using PSIPRED version 3.3 (http://bioinf.cs.ucl.ac.uk/psipred/).

A ubiquitin mutant was generated using the QuikChange Lightning multisite-directed mutagenesis kit (Agilent Technologies), according to the manufacturer's instructions. A Cre active-site mutant was generated using the QuikChange Lightning site-directed mutagenesis kit (Agilent Technologies), according to the manufacturer's instructions.

### Modification of the wild-type R388 plasmid.

The modified R388 plasmids used in this study are described in Table S3. The R388 plasmid was modified using recombineering method according to the multicopy plasmid modification protocol (42, 43). The two-step seamless method using the *cm-sacB* selection cassette was used to create precise genetic changes without otherwise altering the plasmid. The *cm-sacB* cassette is used for positive/negative selection; it can be selected either for (by chloramphenicol resistance) or against (sucrose sensitivity). In the first recombineering step, the sequence to be modified is replaced with the *cm-sacB* cassette; the cassette is then replaced with the desired alteration in the second recombineering event.

The bacterial strain containing the defective λ prophage, SW102 (44), and the plasmid containing *cm-sacB* cassette, pEL04, were obtained from NCI—Frederick. PCR products used for homologous recombination contained at each end on average about 200 bp of flanking homology to the desired region on the plasmid. For this purpose, PCR templates were first generated using In-Fusion HD cloning kit. We first made the templates for the second recombineering step by modifying TrwC encoded on the pBAD-ABC vector (see Tables S1 and S2). The templates for the first recombineering step were then prepared by replacing a desired TrwC modification on a relevant pBAD-ABC vector (encoding relevant modified TrwC) with the *cm-sacB* cassette. PCR products were amplified from linearized templates using Phusion high-fidelity DNA polymerase (NEB), digested with DpnI, and purified using the MinElute gel extraction kit (Qiagen). The details of the templates and PCR products are given below.

To generate an R388 plasmid encoding TrwC with a fusion protein between the relaxase (R) and helicase domains (H), R388_R-Ub/Ub^3,13^/mGFP-H, the template for the first recombineering step was generated by replacing the relaxase and the first part of the helicase domain in the pBAD-ABC_His_ plasmid with the *cm-sacB* cassette, generating the pBAD-AB_Cm-SacB-H plasmid. In the first recombineering step, the PCR fragment containing B_Cm-SacB-H was amplified from the pBAD-AB_Cm-SacB-H plasmid and used to modify the wild-type R388. In the second recombineering step, the PCR fragment containing B_R-Ub/Ub^3,13^/mGFP-H was amplified from the pBAD-AB_R-Ub/Ub^3,13^/mGFP-H plasmid and used to modify R388_B_Cm-SacB-H.

To generate a wild-type R388 plasmid encoding TrwC with duplicated domains, four recombineering steps were necessary. The two-step approach, using a PCR product with duplicated domain sequences in the second step, generated the R388 plasmid encoding wild-type TrwC. This is because the recombineering occurs through a fully single-stranded DNA (ssDNA) intermediate of the PCR fragment (45), most likely resulting in recombination of the duplicated domains, generating the wild-type protein.

To generate an R388 plasmid encoding TrwC with duplicated relaxase domains (R) and a fusion protein between, R388_R-Ub/Ub^3,13^/mGFP-TrwC, the following PCR fragments were generated. For the first recombineering step, the PCR fragment encoding B_Cm-SacB-H was amplified from the pBAD-AB_Cm-SacB-H plasmid. For the second step, the PCR fragment encoding B_R-His_6_-Ub/Ub^3,13^/mGFP-H was amplified from the pBAD-AB_R-His_6_-Ub/Ub^3,13^/mGFP-H plasmid. For the third step, the PCR fragment encoding Ub/Ub^3,13^/mGFP-Cm-SacB-H was amplified from the pBAD-AB_R-His_6_-Ub/Ub^3,13^/mGFP-Cm-SacB-H plasmid. For the fourth step, the PCR fragment encoding Ub/Ub^3,13^/mGFP-TrwC was amplified from the pBAD-AB_R-His_6_-Ub/Ub^3,13^/mGFP-TrwC plasmid.

To generate R388 plasmid encoding TrwC with duplicated helicase domains (H) and a fusion protein between, R388_TrwC-Ub/Ub^3,13^/mGFP-H, the following PCR fragments were generated. For the first step, the PCR fragment encoding H-Cm-SacB-B11 was amplified from the pBAD-ABC-Cam-SacB-trwD/virB11 plasmid. TrwD/VirB11 is the protein encoded downstream of TrwC in the wild-type R388 plasmid. For the second step, the PCR fragment encoding H-Ub/Ub^3,13^/mGFP-His_6_-B11 was amplified from the pBAD-ABC-Ub/Ub^3,13^/mGFP-His_6_-trwD/virB11 plasmid. For the third step, the PCR fragment encoding Ub/Ub^3,13^/mGFP-Cm-SacB-trwD/virB11 was amplified from the pBAD-ABC-Ub/Ub^3,13^/mGFP-Cm-SacB-trwD/virB11 plasmid. For the fourth step, the PCR fragment encoding Ub/Ub^3,13^/mGFP-H-His_6_-trwD/virB11 was amplified from the pBAD-AB_R-Ub/Ub^3,13^/mGFP-H-His_6_-trwD/virB11 plasmid.

Electrocompetent SW102 cells were prepared in the following way. One-and-a-half milliliters of SW102 cells grown overnight at 30°C was diluted in 75 ml of LB medium and grown with shaking at 32°C until reaching an optical density at 600 nm (OD_600_) of 0.5. The λ recombination genes were induced by placing the flask into a 42°C shaking water bath for 15 min. The flask was then cooled in the ice-water slurry, and the electrocompetent cells were prepared by washing the cells twice with 40 ml of ice-cold distilled water and resuspending in 200 μl of distilled water. Fifty microliters of cells was electroporated with 60 ng of plasmid and 100 to 150 ng of purified PCR fragment and shaken for 2 h at 30°C in 1 ml of LB. After 2 h, 9 ml of LB medium containing 12.5 μg/ml chloramphenicol (in the case of first recombineering step) or 10 μg/ml trimethoprim (in the case of second recombineering step) was added, and the culture was grown overnight with shaking at 30°C. The following morning, the plasmid was isolated from the culture using the QIAprep Spin miniprep kit (Qiagen) and transformed into electrocompetent Escherichia coli TOP10 cells, and the recombined plasmid was selected on LB agar plates containing chloramphenicol (in the case of first recombineering step) or trimethoprim and 6% sucrose and lacking NaCl (in the case of second recombineering step). Several Cm-resistant and sucrose-sensitive colonies (in the case of first recombineering step) or Cm-sensitive and sucrose- and Tmp-resistant colonies (in the case of second recombineering step) were grown, and recombinant plasmids were isolated and sequenced.

### Cre recombination test.

Prior to the CRAfT experiments described below, Cre recombinase activity for each Cre fusion construct was tested. CSH26Cm::LTL cells (Tc resistant [Tc^r^]; Lang et al. [36]) carrying pBAD-ABC (Amp resistant [Amp^r^]) with or without modified TrwC (as indicated in Results) were grown overnight in LB medium containing tetracycline, ampicillin, and 0.4% glucose. One hundred twenty-five microliters of the overnight culture was pelleted and resuspended in 5 ml of LB medium containing ampicillin. The culture was grown at 37°C until reaching an OD_600_ of 0.6. The cells were then put on ice. Recombinants were selected by plating serial dilutions on LB agar plates containing chloramphenicol, and the total cell counts were determined as the sum of cells growing on tetracycline plates and cells growing on chloramphenicol plates. There were no cells growing on tetracycline and chloramphenicol plates. The recombination frequencies are calculated as recombinants per total amount of cells.

### CRAfT assay.

The Cre fusion reporter assay was adapted from a study by Lang et al. (36). An overnight culture of TOP10 donor cells carrying pRSF-oriT (Km resistant [Km^r^]) and/or pBAD-ABC-T4SS (Amp^r^) with or without modified TrwC (as indicated in Results) was diluted 20× in LB medium containing appropriate antibiotics and grown at 37°C until reaching an OD_600_ of 0.6. The cultures were then induced with 0.08% (vol/vol) arabinose for 1 h. In parallel, an overnight culture of CSH26Cm::LTL recipient cells (Tc^r^) was diluted 40× in LB medium and grown at 37°C. Donors corresponding to an OD of 1 and recipients corresponding to an OD of 0.1 were spun and resuspended in 50 μl of LB. The mixture was pipetted onto filter paper (MF-Millipore membrane, mixed cellulose esters, 0.45 μm) lying on top of an LB plate that was well dried beforehand. The filter was incubated at 37°C for 2.5 h and then recovered into an Eppendorf tube. The cells were washed off the filter by adding LB medium and gently vortexing the tube. Recombinants were selected by plating serial dilutions on LB agar plates containing chloramphenicol, and when pRSF-oriT was present, transconjugants were selected on LB agar plates containing kanamycin and tetracycline. Recipient cell counts were determined as the sum of cells growing on tetracycline plates and cells growing on chloramphenicol plates. No cells grew on tetracycline and chloramphenicol plates. The conjugation and protein translocation frequencies are calculated as the number of transconjugants and recombinants per recipient, respectively.

### TrwC purification.

A culture of E. coli TOP10 cells carrying pBAD-ABC_His_ (Amp^r^) was grown at 37°C from a single colony until reaching an OD_600_ of 0.6. Protein expression was induced with 0.08% (vol/vol) arabinose, and the culture was incubated overnight at 18°C with shaking at 200 rpm. Cells were harvested and resuspended in ice-cold resuspension buffer (50 mM HEPES, 15 mM imidazole [pH 7.8] at 4°C) supplemented with 0.5 mg/ml lysozyme and protease inhibitors (Complete EDTA-free; Roche). After resuspension, the lysate was supplemented with 5 mM MgCl_2_ and 25 U/ml Benzonase (Merck Millipore) and incubated for 15 min on ice. The lysate was then supplemented with 250 mM NaCl and passed through a high-pressure homogenizer (Emulsiflex-C5; Avestin). The cell lysate was clarified by centrifugation at 100,000 × *g* for 30 min at 4°C and applied to a 5-ml HiTrap chelating HP column (GE Healthcare) loaded with cobalt ions and equilibrated with wash buffer (50 mM HEPES, 250 mM NaCl, 20 mM imidazole [pH 7.8] at 4°C). The column was then washed extensively first with wash buffer followed by high-salt buffer A (50 mM HEPES, 500 mM NaCl [pH 7.8] at 4°C) and finally reequilibrated with wash buffer. The protein was eluted with 10 column volumes of linear imidazole gradient (A, wash buffer; B, 50 mM HEPES, 200 mM NaCl, 500 mM imidazole [pH 8.0] at 4°C). The eluted protein was applied to a 5-ml HiTrap SP HP column (GE Healthcare) equilibrated with low-salt buffer (50 mM HEPES, 250 mM NaCl [pH 7.8] at 4°C). The protein was eluted with 10 column volumes of linear salt gradient (A, low-salt buffer; B, 50 mM HEPES, 1 M NaCl [pH 7.8] at 4°C). The eluted protein was further purified by gel filtration using a Superdex 200 10/300 GL column equilibrated in gel filtration buffer (50 mM HEPES, 500 mM NaCl [pH 7.8] at 4°C). The protein concentration was determined by measuring the absorbance at 280 nm and using a molar extinction coefficient calculated from its primary sequence (Expasy [http://expasy.org/tools/protparam.html]). Proteins were supplemented with glycerol to a final concentration of 20% (vol/vol), flash-frozen, and stored at −80°C.

mGFP-TrwC and GA-TrwC (TrwC with additional two residues, glycine and alanine, at its N terminus) were purified as described above for TrwC-His_6_, with the following modifications. pETZt-mGFP-trwC and pETZt-trwC (Km^r^) were used to transform E. coli BL21 Star (DE3) cells. Protein expression was induced with 0.1 mM isopropyl-β-d-thiogalactopyranoside (IPTG). In the case of GA-TrwC, following the ion-exchange step, the N-terminal His_6_-Z tag was cleaved off during an overnight incubation at 4°C with His_6_-tagged TEV protease (1 mg of protease per 30 mg of substrate). The mixture was simultaneously dialyzed into wash buffer B (50 mM HEPES, 500 mM NaCl, 20 mM imidazole [pH 7.8] at 4°C). Cleaved His_6_-Z tag and the protease were removed by rebinding to a cobalt-charged HiTrap chelating HP column equilibrated with wash buffer B. The flowthrough (containing cleaved proteins) was concentrated and further purified by gel filtration, as described above.

### *oriT* nicking assay.

To test DNA nicking by different TrwC constructs, pUC-oriT was mixed with either TrwC-His_6_, GA-TrwC, or mGFP-TrwC and incubated for 30 min at 37°C. The final mixture contained 40 nM pUC-oriT and 40, 80, or 160 nM TrwC, and the final binding buffer contained 30 mM HEPES (pH 7.6) at 25°C, 100 mM NaCl, and 5 mM MgCl. After incubation, the reaction mixtures were supplemented with NuPAGE LDS sample buffer (Thermo Fisher Scientific) and EDTA at a final concentration of 1× and 5 mM, respectively. The samples were heated at 95°C for 5 min and loaded onto a NuPAGE Novex 4 to 12% Bis-Tris gel (Thermo Fisher Scientific). Following SDS-PAGE, the gel was stained with Sypro Ruby protein gel stain (Thermo Fisher Scientific), according to the manufacturer's instructions. TrwC constructs were visualized using a FLA-3000 fluorescent imaging scanner (Fujifilm).

To test its specificity of DNA nicking, TrwC-His_6_ was mixed with either the pUC-oriT or pUC18 plasmid and incubated for 30 min at 37°C. The final mixture contained 50 or 100 nM TrwC-His_6_ and 20 nM plasmid, and the final binding buffer contained 30 mM HEPES (pH 7.6) at 25°C, 100 mM NaCl, 5 mM MgCl_2_, and 4% glycerol. After incubation, the samples were further processed as described above.

### R388 conjugation assay.

An overnight culture of TOP10 donor cells (Sm resistant [Sm^r^]) carrying an R388 plasmid (Tmp resistant [Tmp^r^]) with wild-type or modified TrwC (as indicated in Results) was diluted 20× in LB medium and grown at 37°C until reaching an OD of 0.6. In parallel, an overnight culture of CSH26Cm::LTL recipient cells (Tc^r^) was diluted 30× in LB medium and grown at 37°C. Donors corresponding to an OD of 0.3 and recipients corresponding to an OD of 0.6 were spun and resuspended in 50 μl of LB. This OD ratio corresponded to 3 recipients per donor. The mixture was pipetted onto a filter paper as described for the CRAfT experiments and incubated at 37°C for 1.5 h. The filter was then placed into an Eppendorf tube, and cells were washed off by the addition of LB medium and gentle vortexing. Transconjugants were selected on LB agar plates containing tetracycline and trimethoprim. Donor cell counts were determined with streptomycin and trimethoprim, and recipient cell counts were determined with tetracycline. The conjugation frequencies are calculated as transconjugants per donor.

### Data availability.

All relevant data are available from the authors upon request.

## Supplementary Material

Supplemental material
