# Supplementary material for: Translocation through the Conjugative Type IV Secretion System Requires Unfolding of Its Protein Substrate
Source: J Bacteriol. 2018 Feb 23;200(6):e00615-17. doi: 10.1128/JB.00615-17 (PMC5826034; doi:10.1128/JB.00615-17)
Supplement: Supplemental material [file supp_200_6_e00615-17__index.html]

Translocation through the Conjugative Type IV Secretion System Requires Unfolding of Its Protein Substrate — Supplemental material 

# Translocation through the Conjugative Type IV Secretion System Requires Unfolding of Its Protein Substrate

## Supplemental material

- Supplemental file 1 -

  Fig. S1 (Western blot of donor cells carrying R388 plasmid) and Tables S1 (Plasmids) and S2 (Primers)

  PDF, 816K
