# Supplementary material for: Translocation through the Conjugative Type IV Secretion System Requires Unfolding of Its Protein Substrate
Source: J Bacteriol. 2018 Feb 23;200(6):e00615-17. doi: 10.1128/JB.00615-17 (PMC5826034; doi:10.1128/JB.00615-17)
Supplement: Supplemental material [file JB.00615-17_zjb999094662s1.pdf]

Supplemental material

Figure S1. Western blot of donor cells carrying R388 plasmid encoding wild-type or modified TrwC as indicated detected using self-made polyclonal α-TrwC antibody.

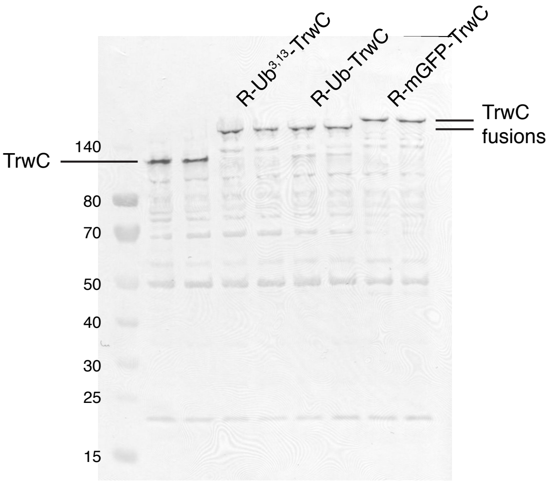

Shown are duplicates for each construct. The vast majority of the expressed TrwC consists of the full-length protein. There are no additional or stronger degradation bands in the R-$Ub^{3,13}$-TrwC construct in comparison to R-Ub-TrwC.

Supplementary table 1. Plasmids used in this study

Plasmids	Description	Source
pRSFDuet-1	Vector carrying two T7 promoters, RSF replicon, *lacI* gene and kanamycin resistance gene (KnR)	Novagen
pRSF-oriT	pRSFDuet-1 vector containing R388 *oriT* sequence and lacking part of *lacI* gene and the first promoter region	This study
pUC18	High copy number cloning vector	Agilent Technologies
pUC-oriT	pUC18 vector containing R388 *oriT* sequence	This study
pBAD-B1-B10Strep	pBADM11 vector containing the genes *trwN/virB1, trwL/virB2, trwM/virB3, trwK/virB4, trwJ/virB5, trwI/virB6, trwH/virB7, trwG/virB8, trwF/virB9*, and *trwE/virB10*, with a Strep tag fused to TrwE C-terminus	(1)
pASK-B1-B10Strep-B11	pASK-IBA3C vector containing the R388 genes *virB1-virB11*, with a Strep tag fused to VirB10 C-terminus	(2)
pBAD-B1-B10Strep-B11	Identical to pBAD-B1-B10Strep, but containing *virB11* downstream of *virB10Strep*	This study
pBAD/MCS	Expression vector carrying *araBAD* promoter, *araC* gene and ampicillin resistance gene (AmpR)	EMBL
pBAD-ABCHis	pBAD/MCS vector containing the R388 genes *trwA*, *trwB*, and *trwC* with a (Gly)3 linker and a hexahistidine tag fused to the TrwC C-terminus	This study
pBAD-ABC-T4SS	Identical to pBAD-B1-B10Strep-B11, but containing the genes *trwA, trwB,* and *trwC* and a consensus *E. coli* rbs sequence upstream of *virB1*; fused to the TrwC C-terminus is a hexahistidine tag separated by a (Gly)3 linker	This study
pBABE-Cre-ER-ERT2	Vector encoding Cre recombinase	(3)
pBAD-AB_Cre-C	Identical to pBAD-ABCHis but containing Cre recombinase fused to the TrwC N-terminus with a (Gly)3Ser(Gly)2 linker	This study
pBAD-AB_R-Cre-H	Identical to pBAD-ABCHis but containing Cre recombinase inserted between the TrwC relaxase (R; aa 1-312) and helixase (H; aa 1-313) domains with (Gly)3SerGly linkers at both sides	This study
pBAD-ABC-Cre	Identical to pBAD-ABCHis but containing (Gly)3-mGFP-(Gly)3-His6 fused to the TrwC C-terminus	This study
pBAD-AB_Cre-C_T4SS	Identical to pBAD-AB_Cre-C but containing a consensus E. coli rbs sequence followed by *virB1-virB11* genes downstream of *trwC* (TrwE has a Strep tag fused to the C-terminus)	This study
pBAD-AB_R-Cre-H_T4SS	Identical to pBAD-AB_R-Cre-H but containing a consensus E. coli rbs sequence followed by *virB1-virB11* genes downstream of *trwC* (TrwE has a Strep tag fused to the C-terminus)	This study
pBAD-ABC-Cre_T4SS	Identical to pBAD-ABC-Cre but containing a consensus E. coli rbs sequence followed by *virB1-virB11* genes	This study

	downstream of *trwC* (TrwE has a Strep tag fused to the C-terminus)	
pBAD-AB_Cre(Y324F)-C	Identical to pBAD-AB_Cre-C but containing Y324F mutation in Cre	This study
pETZt	Expression vector carrying *lacI* gene, kanamycin resistance gene (KnR), and T7 promoter encoding a hexahistidine tag followed by Z tag and TEV protease cleavage site	EMBL
pETZt-TrwC	pETZt vector encoding TrwC with an N-terminal hexahistidine tag followed by Z tag and TEV cleavage site	This study
pETZt-mGFP-TrwC	pETZt vector encoding TrwC with an N-terminal hexahistidine tag followed by Z tag, TEV cleavage site, and mGFP	This study
pFastBacHT-mGFP	A modified pFastBacHTa vector encoding monomeric GFP (the K221L mutation of eGFP; Zacharias et al. 2002)	(4)
pBAD-ABC-mGFP	Identical to pBAD-ABC-Cre but containing mGFP instead of Cre recombinase	This study
pBAD-AB_R-mGFP-H	Identical to pBAD-AB_R-Cre-H but containing mGFP instead of Cre recombinase	This study
pBAD-AB_mGFP-C	Identical to pBAD-AB_Cre-C but containing mGFP instead of Cre recombinase	This study
pET17-Ub	pET17b vector encoding ubiquitin (Ub)	(5)
pET17-UbI3G,I13G	pET17b vector encoding ubiquitin mutant; Ile at amino acid positions 3 and 13 mutated to Gly	This study
pBAD-AB_Ub-C	Identical to pBAD-AB_Cre-C but containing Ub instead of Cre recombinase	This study
pBAD-AB_Ub3,13-C	Identical to pBAD-AB_Ub-C apart from ubiquitin Ile to Gly mutations at amino acid positions 3 and 13	This study
pBAD-AB_Cre-Ub-C	Identical to pBAD-ABCHis but containing Cre-(Gly)3SerGly-Ub-(Gly)3Ser(Gly)2 fused to the TrwC N-terminus	This study
pBAD-AB_Cre-Ub3,13-C	Identical to pBAD-AB_Cre-Ub-C apart from ubiquitin Ile to Gly mutations at amino acid positions 3 and 13	This study
pBAD-AB_Cre-mGFP-C	Identical to pBAD-AB_Cre-Ub-C but containing mGFP instead of Ub	This study
pBAD-AB_Cre-Cre(Y324F)-C	Identical to pBAD-AB_Cre-Ub-C but containing Cre(Y324F) instead of Ub	This study
pBAD-AB_Cre-Ub-C_T4SS	Identical to pBAD-AB_Cre-Ub-C but containing a consensus E. coli rbs sequence followed by *virB1-virB11* genes downstream of *trwC* (TrwE has a Strep tag fused to the C-terminus)	This study
pBAD-AB_Cre-Ub3,13-C_T4SS	Identical to pBAD-AB_Cre-Ub3,13-C but containing a consensus E. coli rbs sequence followed by *virB1-virB11* genes downstream of *trwC* (TrwE has a Strep tag fused to the C-terminus)	This study
pBAD-AB_Cre-mGFP-C_T4SS	Identical to pBAD-AB_Cre-mGFP-C but containing a consensus E. coli rbs sequence followed by *virB1-virB11* genes downstream of *trwC* (TrwE has a Strep tag fused to the C-terminus)	This study

pBAD-AB_Cre-Cre(Y324F)-C_T4SS	Identical to pBAD-AB_Cre-Cre(Y324F)-C but containing a consensus E. coli rbs sequence followed by *virB1-virB11* genes downstream of *trwC* (TrwE has a Strep tag fused to the C-terminus)	This study
pBAD-AB_R-Ub-H	Identical to pBAD-ABCHis but containing (Gly)3Ser(Gly)3-Ub-(Gly)3SerGly inserted between the TrwC relaxase (R; aa 1-312) and helixase (H; aa 313-966) domains	This study
pBAD-AB_R-Ub3,13-H	Identical to pBAD-AB_R-Ub-H apart from ubiquitin Ile to Gly mutations at amino acid positions 3 and 13	This study
pBAD-AB_R-Ub-C	pBAD/MCS vector containing the genes *trwA, trwB*, and modified *trwC*; TrwC containes a duplicated relaxase domain (R; aa 1-305) followed by (Gly)3-His6-GlySer(Gly)3-Ub-(Gly)3SerGly at its N-terminus and the second relaxase domain lacks the first methionine	This study
pBAD-AB_R-Ub3,13-C	Identical to pBAD-AB_R-Ub-C apart from ubiquitin Ile to Gly mutations at amino acid positions 3 and 13	This study
pBAD-AB_R-mGFP-C	Identical to pBAD-AB_R-Ub-C but containing mGFP instead of ubiquitin	This study
pBAD-ABC-Ub-H	pBAD/MCS vector containing the genes *trwA, trwB*, and modified *trwC*; TrwC containes (Gly)3Ser(Gly)3-Ub-(Gly)3SerGly followed by a duplicated helicase domain (H; aa 313-966) and a hexahistidine tag at its C-terminus	This study
pBAD-ABC-Ub3,13-H	Identical to pBAD-ABC-Ub-H apart from ubiquitin Ile to Gly mutations at amino acid positions 3 and 13	This study
pBAD-ABC-mGFP-H	Identical to pBAD-ABC-Ub-H but containing mGFP instead of ubiquitin	This study
pEL04	Vector containing the *cm-sac*B cassette to be used for positive/negative selection in recombineering. Positive selection using chloramphenicol, and negative selection against the s*acB* gene using sucrose.	NCI at Frederic k
R388_R-Ub-TrwC	R388 plasmid encoding modified TrwC; TrwC containes a duplicated relaxase domain (R; aa 1-305) followed by (Gly)3-His6-GlySer(Gly)3-Ub-(Gly)3SerGly at its N-terminus and the second relaxase domain lacks the first methionine	This study
R388_R-Ub3,13-TrwC	Identical to R388_R-Ub-TrwC apart from ubiquitin Ile to Gly mutations at amino acid positions 3 and 13	This study
R388_R-mGFP-TrwC	Identical to R388_R-Ub-TrwC but containing mGFP instead of ubiquitin	This study
R388_TrwC-Ub-H	R388 plasmid encoding modified TrwC; TrwC containes (Gly)3Ser(Gly)3-Ub-(Gly)3SerGly followed by a duplicated helicase domain (H; aa 313-966) and a hexahistidine tag at its C-terminus	This study
R388_TrwC-Ub3,13-H	Identical to R388_TrwC-Ub-H apart from ubiquitin Ile to Gly mutations at amino acid positions 3 and 13	This study

R388_TrwC-mGFP-H	Identical to R388_TrwC-Ub-H but containing mGFP instead of ubiquitin	This study
R388_R-Ub-H	R388 plasmid encoding modified TrwC; TrwC contains (Gly)3Ser(Gly)3-Ub-(Gly)3SerGly inserted between the TrwC relaxase (R; aa 1-312) and helixase (H; aa 313-966) domains	This study
R388_R-Ub3,13-H	Identical to R388_R-Ub-H apart from ubiquitin Ile to Gly mutations at amino acid positions 3 and 13	This study
R388_R-mGFP-H	R388 plasmid encoding modified TrwC; TrwC contains mGFP inserted between the TrwC relaxase (R; aa 1-312) and helixase (H; aa 1-313) domains with (Gly)3SerGly linkers at both sides	This study

Supplementary table 2. Primers used in this study. Restriction enzyme sites are shown in bold.

Name of the primers	Sequence of the primers	Name of the construct cloned using the primers	Description
BssHII-oriT fwd<br>BssHII-oriT rev	TA**GCGCGC**CACTCATTTTCTGCATCATTGTAGCACCATC<br>TA**GCGCGC**CACCGCCTCGTCCTCCAAAAGTG	pRSF-oriT	primers used to amplify *oriT* from R388 plasmid
HindIII-oriT fwd<br>SacI-oriT rev	TAT**AAGCTT**CTCATTTTCTGCATCATTGTAGCACCATC<br>TAT**GAGCTC**CCGCCTCGTCCTCCAAAAGTG	pUC-oriT	primers used to amplify *oriT* from pRSF-oriT plasmid
Strep-B11 fwd<br>B11 rev	GCCACCCGCAGTTCGAAAAATAAGG<br>CTCTCTTATTTTTCGCCCTGCAGGTCGACCTCG	pBAD-B1-B10Strep-B11	primers used to amplify *Strep-virB11* from pASK-B1-B10Strep-B11 plasmid
NcoI-TrwA fwd<br>TrwC-His rev	AGGAATTAACCATGGCACTAGGCGACCCCATC<br>GATGATGATGATGATGACCGCCACCCCTTCCGGCCTCCATGC	pBAD-ABCHis	primers used to amplify *trwABC* while adding a hexahistidine tag to TrwC C-terminus
pBAD/MCS fwd<br>pBAD/MCS rev	ATCATCATCATCATCATTAATGAGCTCCGTCGACAAGCTTGC<br>CCATGGTTAATTCCTCCTGTTAGCC	pBAD-ABCHis	primers used to amplify pBAD/MCS vector for inserting *trwABC* and adding a hexahistidine tag to TrwC C-terminus
TrwA fwd<br>His-rbs rev	GAGGAATTAACCATGGCACTAGG<br>TCTGCCAGTGCCATGGTTAATTCCTCCTTGTCGACGGAGCTCATTAATGATG	pBAD-ABC-T4SS	primers used to amplify *trwABCHis* from pBAD-ABCHis plasmids (including all plasmids containing modified TrwC) and adding rbs (red) downstream of *trwCHis*
C-mGFP fwd<br>C-mGFP rev	AGGGGTGGCGGTGTGAGCAAGGGCGAGGAGC<br>TGATGATGATGCCCGCCTCCCTTGTACAGCTC	pBAD-ABC-mGFP	primers used to amplify *mGFP* from pFastBacHT-mGFP plasmid
mGFP-His fwd<br>mGFP-TrwC rev	CGGGCATCATCATCATCATCATTAATGAGC<br>CACACCGCCACCCCTTCC	pBAD-ABC-mGFP	primers used to linearize pBAD-ABCHis plasmid
mGFP int fwd<br>mGFP int rev	GGCGGTGGGAGCGGTGTGAGCAAGGGCGAG<br>GCCGCTGCCACCTCCCTTGTACAGCTCGTCC	pBAD-AB_R-mGFP-H	primers used to amplify *mGFP* from pBAD-ABC-mGFP plasmid

mGFP-H fwd<br>mGFP-R rev	GGAGGTGGCAGCGGCATGCCTTCCGACGAGG<br>ACCGCTCCCACCGCCGAATGAATGGGCCTGTTTTTCTG	pBAD-AB_R-mGFP-H	primers used to linearize pBAD-ABCHis plasmid
N-mGFP fwd<br>N-mGFP rev	GACTATCTAATGGTGAGCAAGGGCGAGGAGC<br>CATACCGCCGCTACCGCCTCCCTTGTACAGCTC	pBAD-AB_mGFP-C	primers used to amplify *mGFP* from pBAD-ABC-mGFP plasmid
mGFP-TrwC fwd<br>mGFP-TrwB rev	GGTAGCGGCGGTATGCTCAGTCACATGGTATTGACC<br>CACCATTAGATAGTCCCCTCAACAAAGG	pBAD-AB_mGFP-C	primers used to linearize pBAD-ABCHis plasmid
Ub I3G<br>Ub I13G	CAGGGTCTTCACGAAGCCCTGCATATGTATATCTCC<br>CTCCACTTCGAGAGTGCCGGTCTTACCAGTCAG	pET17-UbI3G,I13G	primers for multi site-directed mutagenesis that incorporate ubiquitin I3G and I13G mutations (underlined)
Ub int fwd<br>Ub int rev	GGCGGTGGGAGCGGTGGGGGCATGCAGATCTTCGTGAAGACCCTGAC<br>GCCGCTGCCACCTCCCCCACCTCTGAGACGGAGG	pBAD-AB_R-Ub-H	primers used to amplify Ub sequence from pET17-Ub plasmid
Ub-H fwd<br>Ub-R rev	GGAGGTGGCAGCGGCATGCCTTCCGACGAGG<br>ACCGCTCCCACCGCCGAATGAATGGGCCTGTTTTTCTG	pBAD-AB_R-Ub-H	primers used to linearize pBAD-ABCHis plasmid
Ub3,13 int fwd<br>Ub int rev	GGCGGTGGGAGCGGTGGGGGCATGCAGGGCTTCGTGAAGACCCTGAC<br>GCCGCTGCCACCTCCCCCACCTCTGAGACGGAGG	pBAD-AB_R-Ub3,13-H	primers used to amplify UbI3G,I13G sequence from pET17-UbI3G,I13G plasmid
Ub-H fwd<br>Ub-R rev	GGAGGTGGCAGCGGCATGCCTTCCGACGAGG<br>ACCGCTCCCACCGCCGAATGAATGGGCCTGTTTTTCTG	pBAD-AB_R-Ub3,13-H	primers used to linearize pBAD-ABCHis plasmid
N-Ub fwd<br>N-Ub rev	ATCTAATGCAGATCTTCGTGAAGACCCTGAC<br>GCCGCTACCGCCTCCCCCACCTCTGAGACGGAGG	pBAD-AB_Ub-C	primers used to amplify Ub sequence from pET17-Ub plasmid
Ub-TrwC fwd<br>Ub-TrwB rev	GGAGGCGGTAGCGGCGGTATGCTCAGTCACATGGTATTGACC<br>AGATCTGCATTAGATAGTCCCCTCAACAAAGG	pBAD-AB_Ub-C	primers used to linearize pBAD-ABCHis plasmid
N-Ub3,13 fwd<br>N-Ub rev	ATCTAATGCAGGGCTTCGTGAAGACC<br>GCCGCTACCGCCTCCCCCACCTCTGAGACGGAGG	pBAD-AB_Ub3,13-C	primers used to amplify UbI3G,I13G sequence from pET17-UbI3G,I13G plasmid
Ub-TrwC fwd<br>Ub3,13-TrwB rev	GGAGGCGGTAGCGGCGGTATGCTCAGTCACATGGTATTGACC<br>AGCCCTGCATTAGATAGTCCCCTCAACAAAGG	pBAD-AB_Ub3,13-C	primers used to linearize pBAD-ABCHis plasmid
C-Cre fwd<br>NC-Cre rev	GGTTCCAATTTACTGACCGTACACC	pBAD-ABC-Cre	primers used to amplify Cre sequence

	CGCCTCCATCGCCATCTTCCAGCA<br>G		from pBABE-Cre-ER-<br>ERT2 plasmid
Cre-His<br>fwd<br>Cre-TrwC<br>rev	ATGGCGATGGAGGCGGGCATCAT<br>CATC<br>CAGTAAATTGGAACCGCCACCCCT<br>TCCG	pBAD-<br>ABC-Cre	primers used to<br>amplify the portion of<br>the pBAD-ABC-mGFP<br>plasmid lacking mGFP
Cre int<br>fwd<br>Cre int<br>rev	GGTTCCAATTTACTGACCGTACAC<br>C<br>GCCACCTCCATCGCCATCTTCCAG<br>CAG	pBAD-<br>AB_R-Cre-<br>H	primers used to<br>amplify Cre sequence<br>from pBABE-Cre-ER-<br>ERT2 plasmid
Cre-H fwd<br>Cre-R rev	GGCGATGGAGGTGGCAGCGGCAT<br>G<br>CAGTAAATTGGAACCGCTCCCACC<br>GCCGAATG	pBAD-<br>AB_R-Cre-<br>H	primers used to<br>amplify the portion of<br>the pBAD-AB_R-<br>mGFP-H plasmid<br>lacking mGFP
N-Cre fwd<br>NC-Cre<br>rev	CTAATGTCCAATTTACTGACCGTAC<br>ACC<br>CGCCTCCATCGCCATCTTCCAGCA<br>G	pBAD-<br>AB_Cre-C	primers used to<br>amplify Cre sequence<br>from pBABE-Cre-ER-<br>ERT2 plasmid
Cre-TrwC<br>fwd<br>Cre-TrwB<br>rev	ATGGCGATGGAGGCGGTAGCGGC<br>GGTATG<br>TAAATTGGACATTAGATAGTCCCCT<br>CAACAAAGG	pBAD-<br>AB_Cre-C	primers used to<br>amplify the portion of<br>the pBAD-AB_mGFP-<br>C plasmid lacking<br>mGFP
Cre<br>Y324F<br>sense<br>Cre<br>Y324F<br>antisense	CCAATGTAAATATTGTCATGAACTT<br>TATCCGTAACCTGG<br>CCAGGTTACGGATAAAGTTCATGA<br>CAATATTTACATTGG	pBAD-<br>AB_Cre(Y3<br>24F)-C	primers for site-<br>directed mutagenesis<br>that incorporate Cre<br>Y324F mutation<br>(underlined)
TrwC fwd<br>TrwC rev	CATGCTCAGTCACATGGTATTGAC<br>C<br>ATCTCATTACCTTCCGGCCTCCAT<br>GC	pETZt-<br>TrwC	primers used to<br>amplify *trwC* from<br>pBAD-ABCHis<br>plasmid
pETZt fwd<br>TrwC-<br>pETZt rev	GGAAGGTAATGAGATCCGGCTGCT<br>AACAAAGC<br>ATGTGACTGAGCATGGCGCCCTGA<br>AAATAAAGATTC	pETZt-<br>TrwC	primers used to<br>linearize pETZt vector
mGFP<br>fwd<br>TrwC rev	GCCATGGTGAGCAAGGGCGAG<br>ATCTCATTACCTTCCGGCCTCCAT<br>GC	pETZt-<br>mGFP-<br>TrwC	primers used to<br>amplify *mGFP-trwC*<br>from pBAD-<br>AB_mGFP-C plasmid
pETZt fwd<br>mGFP-<br>pETZt rev	GGAAGGTAATGAGATCCGGCTGCT<br>AACAAAGC<br>CTTGCTCACCATGGCGCCCTGAAA<br>ATAAAGATTC	pETZt-<br>mGFP-<br>TrwC	primers used to<br>linearize pETZt vector
Cre-Ub<br>fwd<br>TrwC int<br>rev	GGGAGCGGTGGGGGCATGCAGAT<br>CTTCGTGAAGACCCTG<br>GCATAGGCATGATCGACGTGC	pBAD-<br>AB_Cre-<br>Ub-C	primers used to<br>amplify Ub-TrwC<br>sequence from pBAD-<br>AB_Ub-C plasmid

TrwC int fwd<br>linker-Cre rev	CGATCATGCCTATGCGACGAC<br>GCCCCCACCGCTCCCACCATCGC CATCTTCCAGCAG	pBAD-AB_Cre-Ub-C<br>pBAD-AB_Cre-Ub3,13-C<br>pBAD-AB_Cre-mGFP-C<br>pBAD-AB_Cre-Cre(Y324F)-C	primers used to linearize pBAD-AB_Cre-C plasmid
Cre-Ub3,13 fwd<br>TrwC int rev	GGGAGCGGTGGGGGCATGCAGG GCTTCGTGAAGACC<br>GCATAGGCATGATCGACGTGC	pBAD-AB_Cre-Ub3,13-C	primers used to amplify Ub3,13-TrwC sequence from pBAD-AB_Ub3,13-C plasmid
Cre-mGFP fwd<br>TrwC int rev	GGGAGCGGTGGGGGCGTGAGCAA GGGCGAGGAGC<br>GCATAGGCATGATCGACGTGC	pBAD-AB_Cre-mGFP-C	primers used to amplify mGFP-TrwC sequence from pBAD-AB_mGFP-C plasmid
Cre-Cre fwd<br>TrwC int	GGGAGCGGTGGGGGCTCCAATTT ACTGACCGTACACCAAAATTTGC<br>GCATAGGCATGATCGACGTGC	pBAD-AB_Cre-Cre(Y324F)-C	primers used to amplify Cre(Y324F)-TrwCsequence from pBAD-AB_Cre(Y324F)-C plasmid
pBAD fwd<br>His-R rev	CGGAAGGTAATGAGCTCCGTCGAC AAGCTTG<br>GTGATGATGGTGATGACCGCCACC TGAACCGCCCTTCTCCC	pBAD-AB_R-Ub-C<br>pBAD-AB_R-Ub3,13-C	primers used to linearize pBAD-ABCHis plasmid lacking helicase domain and the hexahistidine tag
His-Ub fwd<br>linker-Ub rev	CATCACCATCATCACCATGGGAGC GGTGGGGGCATG<br>GCCGCTGCCACCTCCCCCACCTCT GAGACGGAGGACC	pBAD-AB_R-Ub-C<br>pBAD-AB_R-Ub3,13-C	primers used to amplify Ub/Ub3,13 sequence from pBAD-AB_R-Ub-H plasmid
linker-TrwC fwd<br>TrwC rev 2	GGAGGTGGCAGCGGCCTCAGTCA CATGGTATTGACCCGACAG<br>GCTCATTACCTTCCGGCCTCCATG CC	pBAD-AB_R-Ub-C<br>pBAD-AB_R-Ub3,13-C	primers used to amplify *trwC* from pBAD-ABCHis plasmid
linker-TrwC 2<br>mGFP-His rev	GGAGGTGGCAGCGGCCTCAGTCA CATGGTATTGACC<br>CTCGCCCTTGCTCACGCCCCCACC GCTCCCATG	pBAD-AB_R-mGFP-C	primers used to linearize pBAD-AB_R-Ub-C plasmid lacking ubiquitin sequence
mGFP fwd 2	GTGAGCAAGGGCGAGGAGC<br>GCCGCTGCCACCTCCCTTG	pBAD-AB_R-mGFP-C	primers used to amplify mGFP sequence from pBAD-

mGFP rev 2			AB_R-mGFP-H plasmid
TrwC-linker fwd<br>TrwB rev	CGGAAGGGGCGGTGGGAGCGGTGG<br>CCTCAACAAAGGCCGGTTGC	pBAD-ABC-Ub-H<br>pBAD-ABC-Ub3,13-H	primers used to amplify Ub/Ub3,13-H-pBAD-AB from pBAD-AB_R-Ub/Ub3,13-H plasmid
TrwB fwd<br>TrwC rev 3	CGGCCTTTGTTGAGGGGACTATC<br>CCACCGCCCCTTCCGGCCTCCATG	pBAD-ABC-Ub-H<br>pBAD-ABC-Ub3,13-H	primers used to amplify *trwC* from pBAD-ABCHis plasmid
mGFP fwd 2<br>TrwB rev	GTGAGCAAGGGCGAGGAGC<br>CCTCAACAAAGGCCGGTTGC	pBAD-ABC-mGFP-H	primers used to linearize pBAD-AB_R-mGFP-H plasmid lacking the relaxase domain sequence
TrwB fwd<br>mGFP-linker rev	CGGCCTTTGTTGAGGGGACTATC<br>CTCGCCCTTGCTCACGCCCCCACCGCTCCCAC	pBAD-ABC-mGFP-H	primers used to amplify *trwC* from pBAD-ABC-Ub-H plasmid

Supplemental References

1- Low, H. H., Gubellini, F., Rivera-Calzada, A., Braun, N., Connery, S., Dujeancourt, A., Lu, F., Redzej, A., Fronzes, R., Orlova, E. V., and Waksman, G. (2014) Structure of a type IV secretion system. *Nature* **508**, 550-553

2- Redzej, A., Ukleja, M., Connery, S., Trokter, M., Felisberto-Rodrigues, C., Cryar, A., Thalassinos, K., Hayward, R. D., Orlova, E. V., and Waksman, G. (2017) Structure of a VirD4 coupling protein bound to a VirB type IV

3- Lang, S., Gruber, K., Mihajlovic, S., Arnold, R., Gruber, C. J., Steinlechner, S., Jehl, M. A., Rattei, T., Frohlich, K. U., and Zechner, E. L. (2010) Molecular recognition determinants for type IV secretion of diverse families of conjugative relaxases. *Molecular microbiology* **78**, 1539-1555

4- Trokter, M., Mücke, N., Surrey, T. (2012) Reconsitution of the human cytoplasmic dynein complex. *Proc. Natl. Acad. Sci.* **109**, 20895-900

5- Hospenthal, M. K., Freund, S. M., Komander, D. (2013) Assembly, analysis and architecture of atypical ubiquitin chains. *Nat. Struct. Mol. Biol.* **20**, 555-65
